# Text-Based Depression Prediction on Social Media Using Machine Learning: Systematic Review and Meta-Analysis

**DOI:** 10.2196/59002

**Published:** 2025-04-11

**Authors:** Doreen Phiri, Frank Makowa, Vivi Leona Amelia, Yohane Vincent Abero Phiri, Lindelwa Portia Dlamini, Min-Huey Chung

**Affiliations:** 1 School of Nursing College of Nursing Taipei Medical University Taipei Taiwan; 2 Department of Information and Communication Technology University of North Carolina Project Lilongwe Malawi; 3 Department of Epidemiology and Environmental Health University at Buffalo New York, NY United States

**Keywords:** depression, social media, machine learning, meta-analysis, text-based, depression prediction

## Abstract

**Background:**

Depression affects more than 350 million people globally. Traditional diagnostic methods have limitations. Analyzing textual data from social media provides new insights into predicting depression using machine learning. However, there is a lack of comprehensive reviews in this area, which necessitates further research.

**Objective:**

This review aims to assess the effectiveness of user-generated social media texts in predicting depression and evaluate the influence of demographic, language, social media activity, and temporal features on predicting depression on social media texts through machine learning.

**Methods:**

We searched studies from 11 databases (CINHAL [through EBSCOhost], PubMed, Scopus, Ovid MEDLINE, Embase, PubPsych, Cochrane Library, Web of Science, ProQuest, IEEE Explore, and ACM digital library) from January 2008 to August 2023. We included studies that used social media texts, machine learning, and reported area under the curve, Pearson *r*, and specificity and sensitivity (or data used for their calculation) to predict depression. Protocol papers and studies not written in English were excluded. We extracted study characteristics, population characteristics, outcome measures, and prediction factors from each study. A random effects model was used to extract the effect sizes with 95% CIs. Study heterogeneity was evaluated using forest plots and *P* values in the Cochran *Q* test. Moderator analysis was performed to identify the sources of heterogeneity.

**Results:**

A total of 36 studies were included. We observed a significant overall correlation between social media texts and depression, with a large effect size (*r*=0.630, 95% CI 0.565-0.686). We noted the same correlation and large effect size for demographic (largest effect size; *r*=0.642, 95% CI 0.489-0.757), social media activity (*r*=0.552, 95% CI 0.418-0.663), language (*r*=0.545, 95% CI 0.441-0.649), and temporal features (*r*=0.531, 95% CI 0.320-0.693). The social media platform type (public or private; *P*<.001), machine learning approach (shallow or deep; *P*=.048), and use of outcome measures (yes or no; *P*<.001) were significant moderators. Sensitivity analysis revealed no change in the results, indicating result stability. The Begg-Mazumdar rank correlation (Kendall τ_b_=0.22063; *P*=.058) and the Egger test (2-tailed *t_34_*=1.28696; *P*=.207) confirmed the absence of publication bias.

**Conclusions:**

Social media textual content can be a useful tool for predicting depression. Demographics, language, social media activity, and temporal features should be considered to maximize the accuracy of depression prediction models. Additionally, the effects of social media platform type, machine learning approach, and use of outcome measures in depression prediction models need attention. Analyzing social media texts for depression prediction is challenging, and findings may not apply to a broader population. Nevertheless, our findings offer valuable insights for future research.

**Trial Registration:**

PROSPERO CRD42023427707; https://www.crd.york.ac.uk/PROSPERO/view/CRD42023427707

## Introduction

### Background

Depression is a highly prevalent mental illness affecting people of various ages worldwide. According to the World Health Organization, more than 350 million people live with depression [1]. Individuals with depressive symptoms encounter challenges in diverse areas of their lives, such as work, relationships, and social interactions [2]. These challenges manifest as sleep problems, diminished energy, loss of interest in daily activities, feelings of worthlessness, trouble focusing, and recurrent suicidal thoughts [3]. In clinical settings, depression is typically identified either through clinical diagnoses or using standardized measurement tools that rely on subjective patient responses. However, both of these methods have limitations. Factors such as the surrounding context, the patient’s mental condition at that time, the relationship between the clinician and patient, the patient’s current emotional state, the patient’s clinical experiences, and memory bias can all affect individuals’ responses [4]. Additionally, people may not be aware of or be ashamed of their depression, which can reduce their willingness to seek professional help. Approximately 70% of individuals avoid seeking professional medical advice during the initial phase of depression [5]. Furthermore, depression diagnosis using conventional approaches based on in-person discussions is expensive both financially and in terms of time; thus, these approaches may not be viable for specific individuals [4]. Therefore, an efficient strategy that can help predict and diagnose depression early in large groups of people would be beneficial.

Social media is regarded as a valuable tool for investigating psychological well-being because it provides access to behaviors, interests, thoughts, and emotions of individuals, all of which may offer insights into their mental health [6]. Depression has received considerable attention in studies on the relationship between mental health disorders and social media [7]. Facebook has more than 2 billion registered users globally, with approximately 1.25 billion active daily users [8]. Twitter (subsequently rebranded X), another popular social network, has approximately 328 million registered users, with approximately 100 million using the platform daily [8]. Various studies used social media textual data to identify and predict depression and other mental health disorders, as textual data provide more meaningful information than visual data [6,9-12]. Text-based prediction involves using text data to anticipate future events, trends, and behavior patterns. In the realm of depression, text-based analysis on social media involves scrutinizing user-generated content from platforms such as Twitter and Facebook to identify signs and symptoms of depression among users. This approach leverages various natural language process techniques and machine learning models. In text-based prediction, the linguistic characteristics of social media text are examined; in addition to the user’s actions on the platform, the language features of words, part-of-speech tags, and n-grams can provide insights into the content and sentiment of web-based conversations [10]. Using social media text to accurately predict depression can facilitate the identification of individuals who require a more comprehensive evaluation; they can then receive relevant resources, support, and treatment [13]. Hence, more research is necessary on using social media textual data for predicting depression.

Based on changes in features such as social media activity, language, and temporal characteristics, accurate statistical models for predicting depression can be developed. Social media textual analysis using traditional statistical methods has limited accuracy because of the unstructured nature of the input data. By contrast, machine learning can effectively examine nonlinear data, rendering it a superior option for analyzing social media data. The use of machine learning to predict depression on social media has increased substantially, as has the range of algorithms used for this purpose [7]. Machine learning can involve either shallow or deep learning, and the success of these methods varies according to the specific task [14]. Machine learning has revolutionized clinical diagnostics, substantially improving prediction and diagnostic capabilities in clinical settings. Therefore, the integration of machine learning techniques in analyzing social media text data holds significant promise for advancing the prediction of depression.

In machine learning-based prediction using textual data, the most relevant features must be extracted from the text to obtain an accurate model. This methodology is regarded as superior to other feature extraction approaches, as it exhibits a higher degree of independence from the system. Consequently, extracting features from text offers considerable flexibility and accuracy [15]. Demographic, language, social media activity, and temporal characteristics are the most common features adopted in prediction studies. Demographic features refer to the age, sex, and geographical location of individuals. A meta-analysis that considered language, social media activity, user demographics, and visual data revealed that demographic features were the most significant predictors of individual characteristics [16]; however, that study assessed the link between social media and personality. Another study also indicated the potential of demographic features for predicting depression using machine learning algorithms; however, social media data were not used [17]. Most social media platforms include user profiles with demographic information that can be used for depression prediction [18]. Thus, assessing the role of demographics in depression prediction is vital.

Language features represent how individuals use words across various categories, such as first-person singular pronouns, words relating to emotions (positive or negative) or sentiment, and depression-related words [19-21]. Studies revealed that people with and without depression have distinct linguistic styles on social media [22], and people who frequently use words related to anxiety are more likely to experience depressive symptoms [22]. A review study also highlighted a relationship between language features and depression; however, the effect was small [20]. Therefore, understanding language features can enhance the accuracy of depression prediction, given their usefulness in identifying depression patterns.

Feature extraction related to social media activity is also valuable for depression prediction. Social media activity features include metrics such as post frequency (daily or weekly), type of content shared, number of words, ratio of posts with URLs, number of friends, number of users following or followed, interactions with friends (likes or comments), and retweet ratio [7,23-25]. Other key activity-related features are the relative volume of posts and reciprocity, which refers to liking and commenting on posts and retweeting and tweeting [23]. The number of likes, comments, and retweets may serve as social attention and stress levels [25]. Studies reported that users who are depressed tend to have a lower volume of posts and reciprocity than users who are not depressed [23,26]. Thus, evaluating the role of social media activity features in depression prediction is crucial.

Furthermore, using temporal features in machine learning can lead to more accurate prediction models for identifying vulnerable individuals on social media. Temporal features refer to various time-related factors, including the user’s status during specific times and the frequency of posts each day. The timing pattern can indicate symptoms of insomnia in depressed individuals. One study reported that temporal features did not contribute to the prediction accuracy of models [27]. In contrast, another study reported that such features improved the accuracy of a model predicting depression based on Twitter data [28]. Limited information is available on the role of temporal features in depression prediction; hence, additional research is required. In summary, studies adopted various features related to social media texts for predicting depression. However, further research is necessary to clarify the usefulness of demographic, social media activity, language, and temporal features in depression prediction.

### Research Problem and Aim

Several studies have reviewed the role of social media textual data in depression prediction [7,9,20,29-31], but the overall evidence remains limited. Previous research often focused on specific features or had a limited scope regarding database searches, and studies included [16,20]. Therefore, in this study, we determine the effectiveness of user-generated social media texts in predicting depression using machine learning. Additionally, we evaluated the influence of demographic, language, social media activity, and temporal features in predicting depression on social media text using machine learning. Our research questions were as follows: How effective are social media texts in predicting depression? Moreover, what is the impact of demographic, language, social media activity, and temporal features in predicting depression using social media texts?

## Methods

### Study Design

We followed the Meta-Analysis of Observational Studies in Epidemiology (MOOSE) guidelines [32] (Multimedia Appendix 1) and registered our review on PROSPERO (CRD42023427707). There were no deviations from the registered protocol.

### Literature Search

Search terms were selected following the population, prediction factors, and outcome format for conducting systematic reviews for prognostic or prediction studies [33]. Our target population was social media users, prediction factors were prediction terms (ie, machine learning, algorithms, text mining, and language style), and the outcome was depression. We systematically searched for relevant studies on CINHAL (through EBSCOhost), PubMed, Scopus, Ovid MEDLINE, Embase, PubPsych, Cochrane Library, Web of Science, ProQuest, IEEE Explore, and ACM digital library. Multimedia Appendix 2 displays the search strings used in the study. Studies published from January 2008 to August 2023 were retrieved. This date range was selected because research on social media only gained prominence from 2008 onwards [7]. Additional relevant studies were identified by manually searching the reference lists of the included studies and other review studies. Authors of the relevant studies that could not be accessible were contacted through email. Endnote version 20 was used to screen the searched studies [33].

### Inclusion and Exclusion Criteria

We included studies that (1) used social media texts to investigate depression, (2) used machine learning, and (3) reported effect sizes (area under the curve, Pearson *r*, and specificity and sensitivity or data used to impute these values) for depression prediction. We excluded protocol papers and studies that were not written in English.

### Screening and Selection of Relevant Studies

Two independent researchers (DP and FM) screened and selected the relevant studies by the inclusion and exclusion criteria. A third researcher (YAVP) was consulted to resolve disagreements. Duplicates were removed, and the remaining studies were evaluated through title and abstract screening. Thereafter, the full texts of potentially eligible studies were screened to identify relevant studies for further analysis.

### Data Extraction

Two independent researchers (DP and FM) extracted the following information from the included studies: (1) study characteristics (publication year, sample size, and data points), (2) population characteristics (demographics and social media platform), (3) outcome measures (eg, Beck Depression Inventory [BDI], Center for Epidemiologic Studies Depression Scale [CES-D], Patient Health Questionnaire-9 [PHQ-9], Depression, Anxiety, and Stress Scales-21 items) or diagnostic framework (eg, *Diagnostic and Statistical Manual of Mental Disorders*), and (4) prediction factors (prediction features, algorithm models, and predictor values [area under the curve, Pearson *r*, and specificity and sensitivity or data used to impute these values]). For studies that reported area under the receiver operating characteristic curve statistics, we first converted the values to Cohen *d*, which were then converted into *r* [34,35]. Furthermore, for studies that provided sufficient information to compute sensitivity and specificity, we used this information to calculate odds ratios [36], which were then converted into *r* values by using Comprehensive Meta-Analysis (version 3; Biostat, Inc). Effect sizes, such as Pearson *r,* allow for comparison across different studies and contexts, contributing to a more general understanding of the relationship between social media text, features, and depression [37]. This is particularly important in meta-analyses, where combining and comparing findings from studies with varying methodologies and populations is crucial [38]. Effect sizes provide a standardized way to do this, making it easier to draw broader conclusions. Furthermore, in many studies, particularly those included in our review, predictive performance metrics are not consistently measured, making it challenging to compare these metrics. By focusing on effect sizes, we ensure that we can consistently assess and compare the core findings related to depression prediction.

The overall effect size was included for studies testing models that incorporated a set of features. In addition, for studies that compared the depression prediction performance of several models based on the same features but employed different algorithms, we extracted the effect size of the model with the optimal performance. Finally, the highest effect size was extracted for studies that did not reveal the effect size of each analyzed feature [8]. Any disagreement between the 2 researchers was resolved by consultation with a third reviewer (VLA).

### Data Analysis

Comprehensive Meta-Analysis version 3 software was used to run the meta-analysis. We adopted a random effects model to assess the uncertainty caused by variations between studies and report the pooled effect size from each study [39]. We used Pearson *r* to determine the relationship between social media texts and depression. Pearson *r* is classified into small (*r*=0.1), medium (*r*=0.3), and large (*r*=0.5) effect sizes [40]. Article heterogeneity was determined using (1) the chi-square *Q* statistic, where *P*<.05 indicates significant heterogeneity, and (2) the *I*^2^ statistic, which represents the extent of variation. An *I*^2^ value of 0% indicates the absence of heterogeneity, and the *I*^2^ values of 1% to 25%, 25% to 75%, and more than 75% indicate low, moderate, and high heterogeneity, respectively [41]. The features adopted in the reviewed studies were grouped into demographic, language, social media activity, and temporal features to assess their overall effect on depression prediction. In addition, we performed a moderator analysis with a random effects model to identify the source of heterogeneity [42]. We included the following groups: social media platform (public and private) [16,43], machine learning (shallow and deep), model validation (10-fold and other), participant sample size (<1000 and ≥1000), data points (<100,000 and ≥100,000), publication (journal and proceedings), features (single and multiple), and use of outcome measures (yes and no). Finally, we conducted a sensitivity analysis to determine the stability of the results [42]. We used a leave-one-out method to examine the effect of each study on the overall effect.

### Publication Bias

We generated a funnel plot to examine the presence of publication bias in the included studies [44]. Statistical evaluation was performed using the Begg-Mazumdar rank correlation and Egger tests [45]. A *P* value of <.05 denotes the presence of significant publication bias. For studies with publication bias, we calculated the adjusted estimated effect sizes by using Duval and Tweedie trim and fill test, with consideration of the potential effect of missing studies [46].

## Results

### Paper Selection Protocol

**Figure 1:**
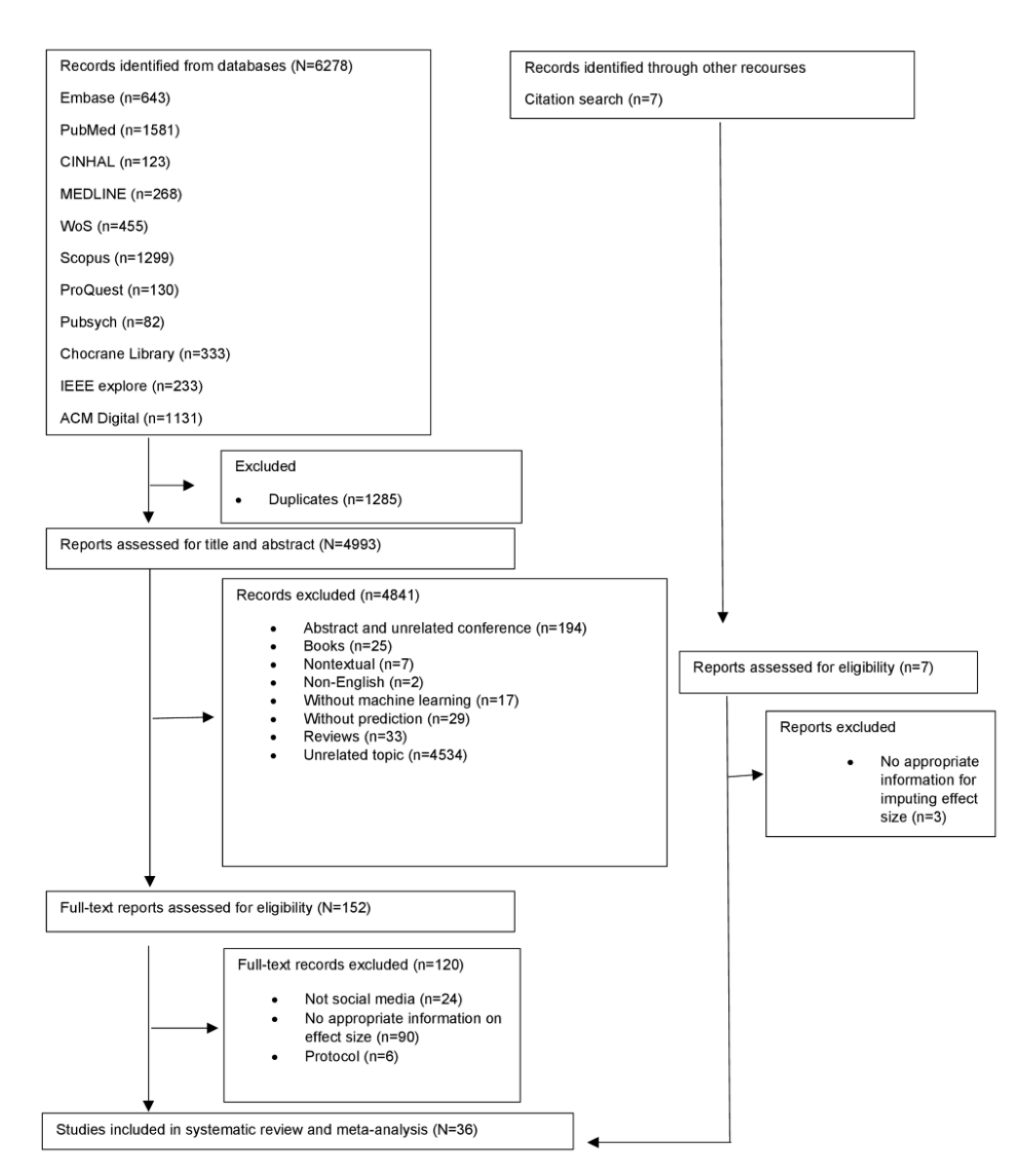
PRISMA (Preferred Reporting Items for Systematic Reviews and Meta-Analyses) flow diagram of study screening and selection. WoS: Web of Science.

The flowchart of study screening and selection is presented in Figure 1. The electronic database search yielded 6278 studies, from which we excluded 1285 duplicates. We screened the abstracts and titles of 4993 studies and excluded 4841 studies that did not meet the eligibility criteria. We conducted full-text screening on 152 eligible studies and excluded studies that did not report social media use (n=24), were protocols (n=6), and had incomplete information to compute effect sizes (n=90). The remaining 32 studies were included in our meta-analysis. Search by citation yielded seven studies, three of which were excluded due to the lack of appropriate information for computing the effect size. The remaining 4 studies were added to the 32 studies, yielding 36 for analysis. Multimedia Appendix 3 provides a list of the included studies.

### Characteristics of Included Studies

The revised PRISMA (Preferred Reporting Items for Systematic Reviews and Meta-Analyses) checklist is provided in Multimedia Appendix 4, and the characteristics of the included studies are outlined in Multimedia Appendix 5. Table 1 summarizes the key information of the included studies. All of the included studies were published between 2013 and 2023; 12 were published in journals and 24 in conference proceedings. Eighteen studies reported the sample size, ranging from 50 to 344,657 (total: 3,969,013). Seven of these studies reported the sex distribution of participants (total participants: 24,502) with 58% identified as females while the remaining 11 papers did not provide information on the sex distribution of the participants. Twitter (n=18, 50%) was the most common platform investigated, followed by Facebook (n=7, 19.4%), Reddit (n=5, 13.9%), Sina Weibo (n=2, 5.6%), Instagram (n=2, 5.6%), Vkontakte (n=2, 5.6%), and Live Journal (n=1, 2.8%). Regarding the machine learning approach, 25 studies adopted shallow learning algorithms (eg, support vector machine, BayesNet, random forest, linear regression, k-nearest neighbor, naïve Bayes, and decision tree), whereas 11 studies used deep learning algorithms (eg, deep neural network, long short-term memory, and convolutional neural network). Eight studies applied 10-fold cross-validation, whereas 9 studies used other types of validation, namely leave-one-out, hold-out, and binary classification validation, as well as 4-fold, 5-fold, and 20-fold cross-validation. Sixteen studies used outcome measures or diagnostic frameworks (Depression, Anxiety, and Stress Scales, CES-D, PHQ-9, PHQ-8, BDI, and *Diagnostic and Statistical Manual of Mental Disorders*), whereas 20 used participants’ diagnostic statements (eg, “I was diagnosed with depression”) or did not provide relevant information. Language features (n=17, 85%) were the most commonly examined features, followed by social media activity (n=8, 40%), temporal (n=4, 20%), and demographic (n=3, 15%) features.

**Table 1:** Characteristics of the included studies (total data points: N=86,324,971).

Characteristics			Studies, n (%)		Participants, n (%)
Data points			29 (80.6)		—^a^
Participants sample size			18 (50)		396,901 (100)
**Sex**			7 (44.4)		24,502 (6.2)
	Male	—		10,286 (42)	
	Female	—		14,216 (58)	
**Social media platform**			36 (100)		—
	Twitter	18 (50)		—	
	Facebook	7 (19.4)		—	
	Reddit	5 (13.9)		—	
	Sina Weibo	2 (5.6)		—	
	Instagram	2 (5.6)		—	
	Vkontakte	2 (5.6)		—	
	LiveJournal	1 (2.8)		—	
**Machine learning approach**			36 (100)		—
	Shallow	25 (69.4)		—	
	Deep	11 (30.6)		—	
**Model validation**			17 (47.2)		—
	10-fold	8 (47.1)		—	
	Other validations^b^	9 (52.9)		—	
**Use of outcome measures**			36 (100)		—
	Yes	16 (44.4)		—	
	No	20 (55.6)		—	
**Publication type**			36 (100)		—
	Journal	12 (33.3)		—	
	Proceeding	24 (66.7)		—	
**Features**			20 (55.5)		—
	Demographics	3 (15)		—	
	Social media activity	8 (40)		—	
	Language	17 (85)		—	
	Temporal	4 (20)		—	

^a^Not applicable.

^b^Leave-one-out, hold out, and binary classification validation; 4-fold, 5-fold, and 20-fold cross-validations.

### The Effectiveness of Social Media Text in Predicting Depression

**Figure 2:**
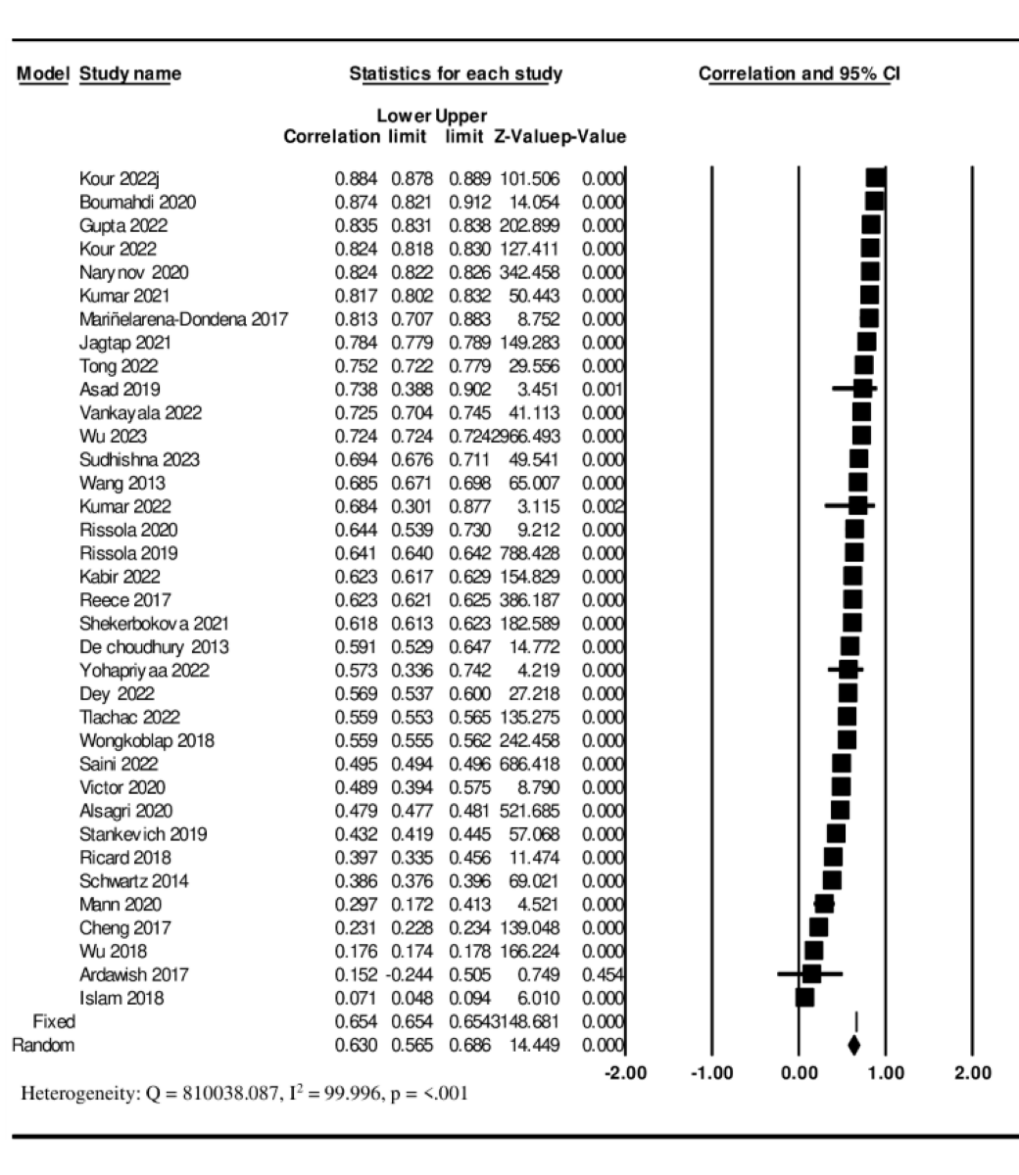
A forest plot of the pooled effect of social media text in predicting depression [47-82].

The 36 included studies [47-82] indicated a large significant overall effect size of *r*=0.630 (95% CI 0.571-0.693), indicating the effectiveness of the user-generated social media text in predicting depression. Significant heterogeneity was observed between the studies (*Q*=810 038.087; *I*^2^=99.996; *P*<.001; Figure 2 [47-82]).

### The Influence of Features in Predicting Depression on Social Media Texts

The findings revealed that all 4 included features; demographic, social media activity, language, and temporal had significant large effect sizes (*r*=0.642; 95% CI 0.489-757; *r*=0.552, 95% CI 0.418-0.663; *r*=0.545, 95% CI 0.434-0.639; and *r*=0.531, 95% CI 0.320-0.693; respectively). A high significant heterogeneity was observed between the studies (*I*^2^=98%-99.9%). Table 2 provides detailed results, and forest plots are presented in Multimedia Appendix 6 [48,50,56,57,59-61,63,64,67,69,70,72,74,78,80-82].

**Table 2:** The influence of features in predicting depression on social media texts.

Feature	Features, n (%)	*r* (95% CI)	*Q*	*I* ^2^	*P* value
Demographic	3 (15)	0.642 (0.489-0.757)	109.732	98.177	<.001
Social media activity	8 (40)	0.552 (0.418-0.663)	96346.650	99.993	<.001
Linguistic	17 (85)	0.545 (0.434-0.639)	782874.071	99.998	<.001
Temporal	4 (20)	0.531 (0.320-0.693)	198014.084	99.998	<.001

### Moderator Analysis

Of all the categories contributing to heterogeneity, only social media platform type, machine learning approach, and use of outcome measures significantly accounted for the observed heterogeneity, with *P* values of <.001, .048, and <.001, respectively (Table 3).

**Table 3:** Moderator analysis.

Category			n (%)		Point estimate (95% CI)		*P* value
**Social media platform**			33 (100)		—^a^		<.001
	Public	28 (84.8)		0.674 (0.618-0.723)		<.001	
	Private	5 (15.2		0.368 (0.166-0.540)		.001	
**Machine learning approach**			36 (100)		—		.048
	Shallow	25 (69.4)		0.584 (0.491-0.663)		<.001	
	Deep	11(30.6)		0.719 (0.610-0.801)		<.001	
**Model validation**			17 (100)		—		.434
	10-fold cross-validation	8 (47.1)		0.522 (0.349-0.660)		<.001	
	Other validations^b^	9 (52.9)		0.602 (0.458-0.715)		<.001	
**Participant sample size**			18 (100)		—		.677
	<1000	11 (61.1)		0.571 (0.423-0.690)		<.001	
	≥1000	7 (38.9)		0.525 (0.330-0.677)		<.001	
**Data points**			29 (100)		—		.627
	<100,000	19 (65.5)		0.650 (0.563-0.723)		<.001	
	≥100,000	10 (34.5)		0.616 (0.486-0.719)		<.001	
**Publication type**			36 (100)		—		.824
	Journal	12 (33.3)		0.637 (0.554-0.707)		<.001	
	Proceedings	24 (66.6)		0.626 (0.565-0.680)		<.001	
**Features**			19 (100)		—		.853
	Single	8 (42.1)		0.552 (0.362-0.698)		<.001	
	Multiple	11 (57.9)		0.536 (0.393-0.652)		<.001	
**Use of outcome measure**			36 (100)		—		<.001
	Yes	16 (44.4)		0.472 (0.381-567)		<.001	
	No	20 (55.6)		0.722 (0.667-769)		<.001	

^a^Not applicable.

^b^Leave-one-out, hold out, and binary classification validation; 4-fold, 5-fold, and 20-fold cross-validation

### Sensitivity Analysis

**Figure 3:**
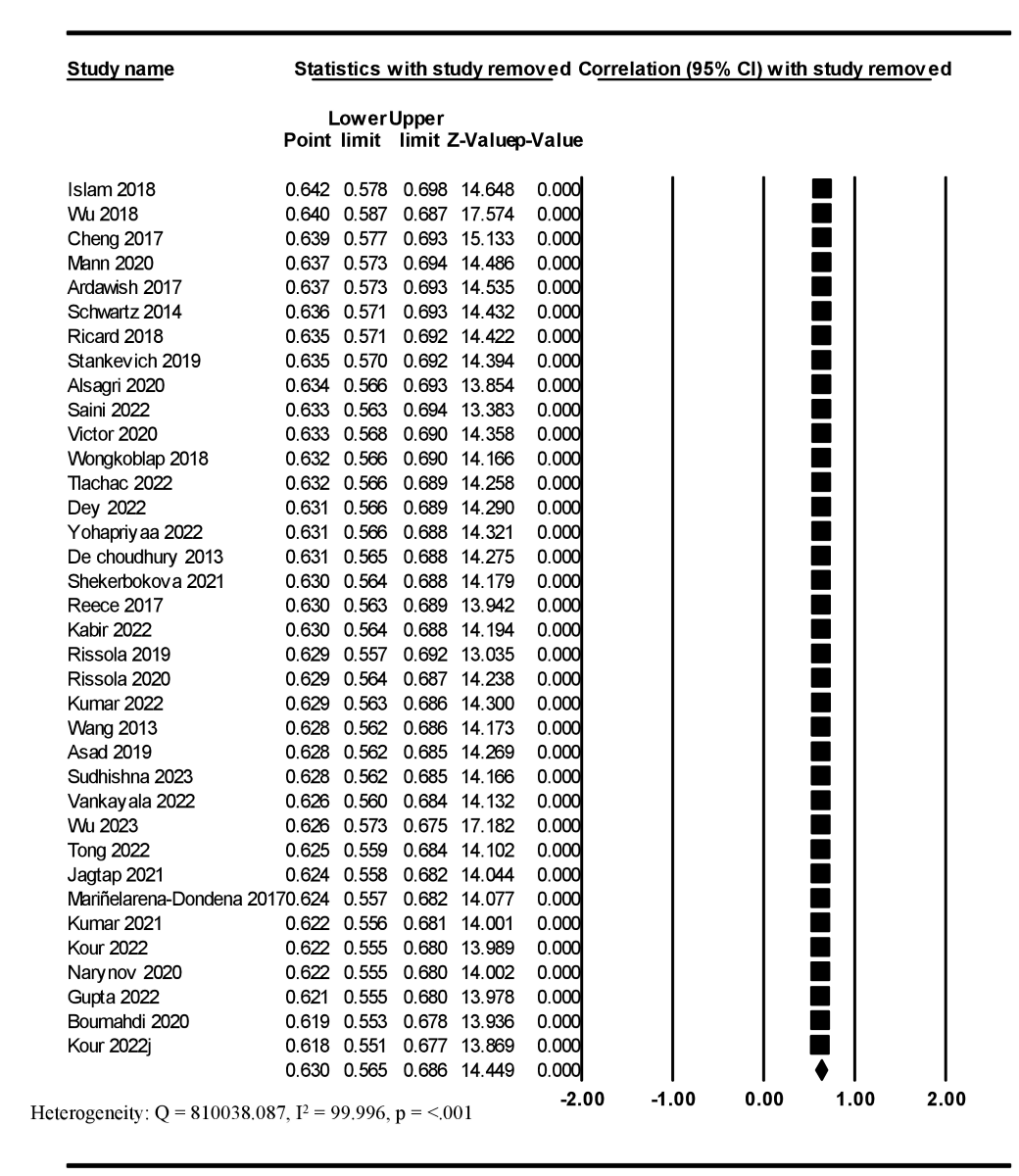
A forest plot of sensitivity analysis [47-82].

The results revealed that the overall effect size did not change (*r*=0.630, 95% CI 0.571-0.693) after performing a leave-one-study-out sensitivity analysis method, and the heterogeneity between the studies was significant (*Q*=810038.087; *I*^2^=99.996; *P*<.001; Figure 3 [47-82]).

### Publication Bias

**Figure 4:**
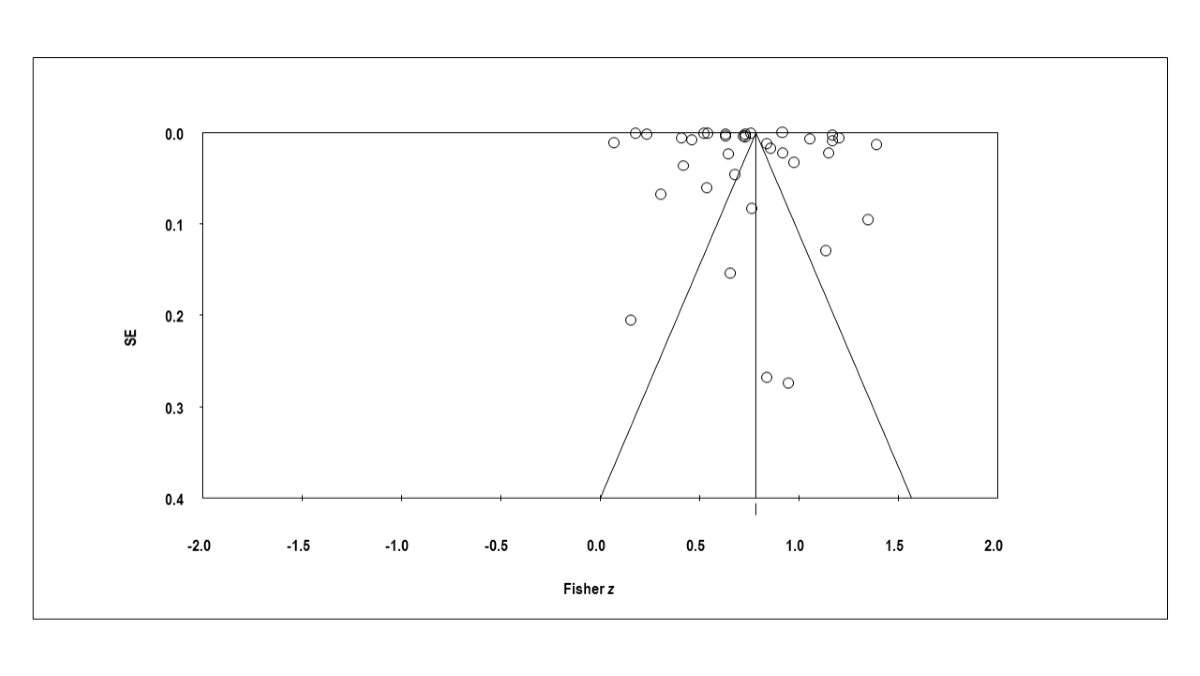
A funnel plot of the publication bias.

Figure 4 presents the funnel plot used to inspect publication bias visually. The nonsignificant results of the Begg–Mazumdar rank correlation test (Kendall τ_b_=0.22063; *P*=.058) and Egger test (2-tailed *t_34_*=1.28696; *P*=.21) indicated the absence of publication bias. The Duval and Tweedie trim and fill test revealed no studies were trimmed [46].

## Discussion

### Principal Findings

This systematic review and meta-analysis examined the effect size of social media texts and the influence of demographics, social media activity, language, and temporal features on depression prediction using machine learning. We observed a significantly large overall effect size for social media texts on depression, indicating the capability of social media texts to predict depression. The demographics, social media activity, language, and temporal features also exhibited significantly large effect sizes, indicating their influence in predicting depression on social media texts. Demographic features had the largest effect size of all the features. Thus, demographic features merit special attention to improve the accuracy of depression prediction models based on social media data. Our results also indicated that the social media platform type (public or private), machine learning approach (shallow or deep), and use of outcome measures (yes or no) were significant moderator variables. Therefore, these factors should be considered in the prediction of depression using social media data. Overall, our findings provide solid evidence that social media texts can predict depression.

### Comparison With Prior Work

The extracted effect size of the social media texts on depression prediction in our study was larger than that reported in a related study (*r=*0.37) between digital traces and psychological well-being [16]. Our results strengthen the growing evidence that social media texts can serve as a valuable means of monitoring and predicting depression. Overall, social media data have considerable potential to advance health research and enhance the capacity to address mental health concerns. Individuals with depression, particularly teenagers and young adults, commonly use social media to express their emotions [83]. Research has revealed that in large prediction models, identifying individuals with depression is more challenging when using social media data than when using electronic health records [84]. However, social media post analysis can yield valuable insights into users’ daily events, activities, and interests [85]. An examination of the language, sentiment, and emotions in these posts can provide information on user behavior, mood, socialization, and opinions [29]. For instance, emotions such as helplessness, worthlessness, and self-hatred in a user’s posts may indicate depression [29]. Therefore, social media is a valuable tool for obtaining a comprehensive overview of a user’s mental health and identifying those with depression.

Our results revealed that demographic, social media activity, temporal, and language features had an influence on depression prediction, with demographic features having the largest effect size. The use of social media textual features in machine learning has proven useful for predicting depression, without any preconceived notions [86]. A related study indicated that the incorporation of demographic data can considerably enhance the accuracy of models linking profile attributes, images, and depression [87]. Similarly, another study reported that demographics are a crucial factor in depression prediction on social media [18]. A study also revealed a significant association between negative emotions and anger and depression among individuals aged 18-25 years on Facebook; however, that study did not use machine learning algorithms [87]. Notably, we analyzed only 3 studies that adopted demographic features, potentially limiting the generalizability of our results. Nevertheless, the results provide strong evidence that demographic data can substantially enhance the prediction value of social media text for depression.

A previous study revealed that the social media activity features on Twitter could predict depression with an accuracy of 69% [24]. Another study, using traditional statistical methods, reported both positive and negative relationships between individual social media activity features and depression on Facebook; individuals with depression exhibited a reduced number of likes and comments, and they posted on their own walls with greater frequency than did their counterparts without depression [26]. Social media activity features can be used to gain valuable insights into individuals’ social media engagement. A reduced level of engagement, particularly a decline in the frequency of their posts, may signify a loss of social connectedness, which has been associated with depressive symptoms [88]. Social media activity features can thus serve as a valuable supplement to self-reported information and clinical evaluations, justifying their inclusion in depression prediction models.

In our study, language features also had a large effect on depression prediction on social media texts. Language features tend to be the most crucial for predicting depression on social media using machine learning. Consistent with our results, a study on depression prediction using social media data and medical records revealed that the strongest predictor of depression was the use of words related to rumination, loneliness, and hostility [89]. Additionally, researchers have suggested that using first-person singular pronouns is associated with depression and suicidal behavior [20,89]. A significant link has also been reported between negative emotions and depression on Facebook; however, the effect was small, and machine learning was not adopted [90]. These findings emphasize the pivotal role of language style in predicting depression on social media.

Our results indicated that temporal features had a large effect on the prediction of depression on social media. By contrast, a related study reported that temporal features did not provide valuable additional user-specific information that could enhance the accuracy of the depression prediction model [27]. The temporal patterns of web-based posts can offer useful insights into the social context and communication styles of individuals [91]; for example, individuals with depression may experience irregular sleep patterns and thus post on social media late at night or early in the morning [92]. Furthermore, consistent with our results, a study that considered temporal measures on Twitter reported that monitoring changes in an individual’s emotional state over time can aid in identifying those who may be experiencing depression [28]. Temporal features may provide the necessary context and help differentiate between a temporary emotional state and a long-term mental health problem. For instance, a single sad post is unlikely to indicate depression, whereas regular sad posts over time suggest a deeper problem that merits attention. Therefore, the inclusion of temporal features can increase the accuracy of depression prediction models based on social media data.

Our findings indicated that the social media platform type, machine learning approach, and use of outcome measures were significant moderators of the relationship between textual features and depression prediction. Most of the studies included in our review focused on public social media platforms, with Twitter being the most common target. Our findings are inconsistent with those of a previous study that reported that the social media platform type had no mediating effect [16]. Although public social media platforms, such as Twitter, cater to diverse user bases and offer varied experiences [93], research has indicated that the use of private social media platforms, such as Facebook, is linked with depressive symptoms, principally because of the loss of real-world social interaction [94]. Further research is thus necessary on the impact of social media platform type on depression prediction.

In our study, the adopted machine learning approach had a significant impact on the relationship between social media text and depression prediction. One study also reported that the accuracy of depression prediction based on textual data depends on the type of model used [9]. Most of the studies included in our analysis adopted shallow machine learning, which involves the use of features such as language style and emotional language as training data for developing a depression prediction model [95]. In general, shallow machine learning models consist of neural networks with only 1 hidden layer. By contrast, deep learning models use neural networks with multiple hidden layers [96]. Shallow learning models typically outperform deep learning in terms of speed and ease of use, but deep learning models can be used to investigate complex phenomena [97,98]. The efficacy of both deep and shallow learning methods depends on the specific application [14]. When limited data are available, training complex neural networks is impossible because these networks require the determination of a large number of parameters from the data; shallow networks are the only option in such cases [14]. Additionally, deep learning models, such as recurrent neural networks, encounter difficulty in identifying the proper context in long sentences [99]. The use of machine learning techniques has contributed considerably to enhancing prediction and diagnostic capabilities in clinical settings [100]. On the basis of unstructured data, this approach can generate insights that can help identify high-risk conditions, facilitating early treatment and improving patient outcomes.

In this review, 20 of the 36 studies did not specify the tools or instruments that were used for depression measurement, or they used subjective patient reports. This could be attributed to many of the studies being performed on public platforms, which is a more cost-effective approach for collecting data from a large sample. A substantial amount of publicly available data are self-reported statements regarding depression diagnoses [13]. Although this approach can yield valuable insights, the findings may have limited accuracy. To ensure reliable and accurate results, the use of survey-based measures, such as CES-D, PHQ-9, or BDI, is paramount, although higher costs would be incurred [101]. The studies included in this review adopted various outcome measures; to elucidate the effectiveness and contribution to the overall prediction accuracy of these tools, a comprehensive examination is imperative.

### Strengths and Limitations

Our study has several strengths. First, to the best of our knowledge, this is the first systematic review and meta-analysis of the effectiveness of user-generated social media texts on depression prediction; our study thus expands depression research. Second, we observed a significantly large overall effect of social media texts on depression prediction, providing strong evidence for the accuracy of social media data in predicting depression. Third, the social media platform, demographic, social media activity, language, and temporal features in this study exhibited a large and more significant influence on depression prediction than similar features in previous studies, providing insights into improving screening and prevention efforts. Fourth, unlike previous reviews, we searched 11 databases, ensuring our search was robust and comprehensive. Fifth, we included 36 studies supporting the reliability of our findings. Previous studies included only 2 [20] and 4 [16] studies, respectively. Sixth, our moderator analysis revealed that the type of machine learning approach affects the accuracy of depression prediction using social media data. Previous reviews did not consider this factor. Seventh, in contrast to previous studies, our sensitivity analysis confirmed the robustness of our findings. This analysis reaffirms the validity of our research and strengthens the credibility of our conclusions.

Despite the strengths and novelty of our study, some limitations should be noted. First, although social media texts are preferred for depression prediction, such data can be more challenging to analyze than survey data because of the considerable variability in the frequency and length of social media posts as well as the likelihood of these variables changing over time. Second, findings based on social media texts cannot be generalized to a wider population because age, income, education, and ethnicity tend not to be proportionately represented in the data. Third, half of the included studies contained insufficient information on the overall effects of the four target features in predicting depression. Fourth, we could not analyze the effects of specific demographics, language, temporal, and social media activity features. Fifth, most of the studies failed to provide information on model validation, a key step for enhancing the accuracy and effectiveness of machine learning. Sixth, our study was limited to English-only studies because reviewing and interpreting studies in unfamiliar languages may lead to misinterpretation and errors in data extraction and analysis. Seventh, many of the included studies were from proceedings, and because most conference papers are not peer-reviewed, the quality of the studies may be in question. Eighth, we could not evaluate the quality of the included studies due to a lack of appropriate guidelines. Ninth, we acknowledge the omission of other keywords like “Reddit” and “classification,” which limits the comprehensiveness of our search strategy. However, considering the novelty of this research area, our findings provide valuable insights that can form the basis of future research. Future studies may explore social media visual data along with specific features within each category (ie, demographic, language, social media activity, and temporal features) and machine learning algorithms for depression prediction on social media. Moreover, it is essential for future research to incorporate a broader range of keywords, including “Reddit” and “classification,” to enhance the search strategy and capture relevant studies effectively.

### Conclusions

Our findings revealed that social media text has a significantly large overall effect on depression prediction using machine learning. Specifically, demographic features had a larger effect size than social media activity, language, and temporal features. Furthermore, the social media platform type, use of outcome measures, and machine learning approach were significant moderators. Our results suggest that social media texts are effective in predicting depression. Social media textual content, particularly demographic features, is thus useful for predicting depression on social media texts. Finally, the social media platform type, machine learning approach used, and use of outcome measures can affect the accuracy of predictions. Our findings may help mental health and psychiatric practitioners identify individuals who require further evaluation; these individuals can receive the necessary resources, support, and treatment.

## Appendix

Multimedia Appendix 1 MOOSE (Meta-Analysis of Observational Studies in Epidemiology) checklist for meta-analyses of observational studies.

Multimedia Appendix 2 Search strategy.

Multimedia Appendix 3 List of included studies.

Multimedia Appendix 4 Revised PRISMA (Preferred Reporting Items for Systematic Reviews and Meta-Analyses) checklist.

Multimedia Appendix 5 Study characteristics.

Multimedia Appendix 6 Forest plots of the influence of demographic, social media activity, language, and temporal features in predicting depression.

## Abbreviations

BDI: Beck Depression Inventory
CES-D: Center for Epidemiological Studies-Depression
MOOSE: Meta-Analysis of Observational Studies in Epidemiology
PHQ-9: Patient Health Questionnaire-9

## Data Availability

Data availability is not applicable to this article as no new data were created or analyzed in this study.
