# Supplementary material for: Text-Based Depression Prediction on Social Media Using Machine Learning: Systematic Review and Meta-Analysis
Source: J Med Internet Res. 2025 Apr 11;27:e59002. doi: 10.2196/59002 (PMC12032503; doi:10.2196/59002)
Supplement: Multimedia Appendix 2 [file jmir_v27i1e59002_app2.docx]

Supplementary Table 1. Search Strategy

| **Database** | **#** |  | **Search terms** |
| --- | --- | --- | --- |
|  |  |  |  |
| EMBASE | 1 | P(F) | Facebook:ti,ab,kw,de OR Twitter:ti,ab,kw,de OR 'social network':ti,ab,kw,de OR 'social media':ti,ab,kw,de |
|  | 2 | P(C) | 'Social media'/exp |
|  | 3 | F(F) | Prediction:ti,ab,kw,de OR predicting:ti,ab,kw,de OR detection:ti,ab,kw,de OR detecting:ti,ab,kw,de OR 'machine learning':ti,ab,kw,de OR 'text mining':ti,ab,kw,de OR 'linguistic style':ti,ab,kw,de OR 'sentiment analysis':ti,ab,kw,de OR 'semantic analysis':ti,ab,kw,de OR 'Natural language processing':ti,ab,kw,de OR algorithms:ti,ab,kw,de |
|  | 4 | F(C) | 'Machine learning'/exp OR 'text mining'/exp OR  'natural language processing'/exp OR  prediction/exp OR detection/exp OR 'sentiment analysis'/exp OR algorithm/exp |
|  | 5 | O(F) | Depression:ti,ab,kw,de OR 'Depressive Symptoms':ti,ab,kw,de OR 'Mood disorder':ti,ab,kw,de |
|  | 6 | O(C) | 'Bipolar disorder'/exp OR 'Major depression'/exp |
|  |  | Filter | English, publication year 2008-2023 |
|  | 7 | All | (#1 OR #2) AND (#3 OR #4) AND (#5 OR #6) |
|  |  |  |  |
| PubMed | 1 | P(F) | ((((Facebook[tw]) OR Twitter[tw]) OR "social network"[tw]) OR "social media"[tw]) |
|  | 2 | P(C) | (("social media"[mh]) OR "social networking"[mh]) |
|  | 3 | F(F) | (((((((((((prediction[tw]) OR predicting[tw]) OR detection[tw]) OR detecting[tw]) OR "machine learning"[tw]) OR "text mining"[tw]) OR " natural language processing"[tw]) OR algorithms [tw]) OR "linguistic style"[tw]) OR "sentiment analysis"[tw]) OR "semantic analysis"[tw]) |
|  | 4 | F(C) | ((((("machine learning"[mh]) OR "natural language processing"[mh]) OR "algorithms"[mh]) OR "sentiment analysis"[mh]) OR "semantics " [mh]) |
|  | 5 | O(F) | (((Depression[tw]) OR "Depressive Symptoms"[tw]) OR "Mood disorder"[tw]) |
|  | 6 | O(C) | (("depressive disorder"[mh]) OR "depression" [mh]) |
|  |  | Filter | English, publication year 2008-2023 |
|  | 7 | All | (#1 OR #2) AND (#2 OR #3) AND (#5 OR #6) |
|  |  |  |  |
| MEDLINE(ovid) | 1 | P(F) | Facebook OR Twitter OR "social network" OR "social media" |
|  | 2 | P(C) | "social media" OR "social networking" |
|  | 3 | F(F) | Prediction OR predicting OR detection OR detecting OR "Machine learning" OR "text mining" OR "linguistic style" OR "sentiment analysis" OR "semantic analysis" OR "natural language processing" OR algorithms |
|  | 4 | F(C) | "machine learning" OR "natural language processing" OR "sentiment analysis" OR "semantics" OR "algorithms" |
|  | 5 | O(F) | Depression OR "Depressive Symptoms" OR "Mood disorder" OR "depressive disorder" |
|  | 6 | O(C) | "depressive disorder" OR "depression" |
|  |  | Filter | English, publication year 2008-2023 |
|  | 7 | All | (#1 OR # 2) AND (#3 OR #4) AND (#5 OR #6) |
|  |  |  |  |
| CINHAL | 1 | P(F) | Facebook OR Twitter OR "social network" OR "social media" |
|  | 2 | P(C) | "social media" OR "social networking" |
|  | 3 | F(F) | Prediction OR predicting OR detection OR detecting OR "Machine learning" OR "text mining" OR "linguistic style" OR "sentiment analysis" OR "semantic analysis" OR "natural language processing" OR algorithms |
|  | 4 | F(C) | "machine learning" OR "natural language processing" OR "sentiment analysis" OR "semantics" OR "algorithms" |
|  | 5 | O(F) | Depression OR "Depressive Symptoms" OR "Mood disorder" OR "depressive disorder" |
|  | 6 | O(C) | "depressive disorder" OR "depression" |
|  |  | Filter | English, publication year 2008-2023 |
|  | 7 | All | (#1 OR # 2) AND (#3 OR #4) AND (#5 OR #6) |
|  |  |  |  |
| ProQuest | 1 | P(F) | Facebook OR Twitter OR "social network" OR "social media" |
|  | 2 | P(C) | "social media" OR "social networking" |
|  | 3 | F(F) | Prediction OR predicting OR detection OR detecting OR "Machine learning" OR "text mining" OR "linguistic style" OR "sentiment analysis" OR "semantic analysis" OR "natural language processing" OR algorithms |
|  | 4 | F(C) | "machine learning" OR "natural language processing" OR "sentiment analysis" OR "semantics" OR "algorithms" |
|  | 5 | O (F) | Depression OR "Depressive Symptoms" OR "Mood disorder" OR "depressive disorder" |
|  | 6 | O(C) | "depressive disorder" OR "depression" |
|  |  | Filter | English, publication year 2008-2023 |
|  | 7 | All | (#1 OR # 2) AND (#3 OR #4) AND (#5 OR #6) AND (#7) |
|  |  |  | (Facebook OR Twitter OR "social network" OR "social media") AND (Prediction OR predicting OR detection OR detecting OR "Machine learning" OR "text mining" OR "linguistic style" OR "sentiment analysis" OR "semantic analysis" OR "natural language processing" OR algorithms) AND (Depression OR "Depressive Symptoms" OR "Mood disorder" OR "depressive disorder") |
|  |  |  |  |
| Scopus | 1 | P(F) | "Facebook" OR "Twitter" OR "social network" OR "social media" |
|  | 2 | P(C) | "social media" OR "social networking" |
|  | 3 | F(F) | "Prediction" OR "predicting" OR "detection" OR "detecting" OR "Machine learning" OR "text mining" OR "linguistic style" OR "sentiment analysis" OR "semantic analysis" OR "natural language processing" OR "algorithms" |
|  | 4 | F(C) | "machine learning" OR "natural language processing" OR "sentiment analysis" OR "semantics" OR "algorithms" |
|  | 5 | O(F) | "Depression" OR "Depressive Symptoms" OR "Mood disorder" OR "depressive disorder" |
|  | 6 | O(C) | "depressive disorder" OR "depression" |
|  |  | Filter | English, publication year 2008-2023 |
|  | 7 | All | (#1) AND (# 2) AND (#3) AND (#4) |
|  |  | query | ( TITLE-ABS-KEY ( "Facebook" OR "Twitter" OR "social network" OR "social media" ) OR TITLE-ABS-KEY ( "social media" OR "social networking" ) AND TITLE-ABS-KEY ( "Prediction" OR "predicting" OR "detection" OR "detecting" OR "Machine learning" OR "text mining" OR "linguistic style" OR "sentiment analysis" OR "semantic analysis" OR "natural language processing" OR "algorithms" ) OR TITLE-ABS-KEY ( "machine learning" OR "natural language processing" OR "sentiment analysis" OR "semantics" OR "algorithms" ) AND TITLE-ABS-KEY ( "Depression" OR "Depressive Symptoms" OR "Mood disorder" OR "depressive disorder" ) OR TITLE-ABS-KEY ( "depressive disorder" OR "depression" ) ) AND PUBYEAR > 2008 AND PUBYEAR < 2023 AND ( LIMIT-TO ( LANGUAGE , "English" ) ) |
| CochraneL | 1 | P(F) | (Facebook OR Twitter OR "social network" OR "social media"):ti,ab,kw |
|  | 2 | P(C) | [mh "social media"] OR [mh "social networking"] |
|  | 3 | F(F) | (Prediction OR predicting OR detection OR detecting OR "Machine learning" OR "text mining" OR "linguistic style" OR "sentiment analysis" OR "semantic analysis" OR "natural language processing" OR algorithms):ti,ab,kw |
|  | 4 | F(C) | [mh "machine learning"] OR [mh "natural language processing"] OR [mh "sentiment analysis"] OR [mh semantics] OR [mh algorithms] |
|  | 5 | O(F) | ("Depression" OR "Depressive Symptoms" OR "Mood disorder" OR "depressive disorder"):ti,ab,kw |
|  | 6 | O(C) | [mh "depressive disorder"] OR [mh "depression"] |
|  |  |  | English, publication year 2008-2023 |
|  | 7 | All | (#1 OR # 2) AND (#3 OR #4) AND (#5 OR #6) |
|  |  |  |  |
| Pubpsych | 1 | P(F) | Facebook OR Twitter OR "social network" OR "social media" |
|  | 2 | P(C) | "social media" OR "social networking" |
|  | 3 | F(F) | Prediction OR predicting OR detection OR detecting OR "Machine learning" OR "text mining" OR "linguistic style" OR "sentiment analysis" OR "semantic analysis" OR "natural language processing" OR algorithms |
|  | 4 | E(C) | "machine learning" OR "natural language processing" OR "sentiment analysis" OR "semantics" OR "algorithms" |
|  | 5 | O(F) | Depression OR "Depressive Symptoms" OR "Mood disorder" OR "depressive disorder" |
|  | 6 | O(C) | "depressive disorder" OR "depression" |
|  |  | Filter | English |
|  | 7 | All | (#1 OR # 2) AND (#3 OR #4) AND (#5 OR #6) |
|  |  |  | (Facebook OR Twitter OR "social network" OR "social media") AND (Prediction OR predicting OR detection OR detecting OR "Machine learning" OR "text mining" OR "linguistic style" OR "sentiment analysis" OR "semantic analysis" OR "natural language processing" OR algorithms) AND (Depression OR "Depressive Symptoms" OR "Mood disorder" OR "depressive disorder") |
|  |  |  |  |
| WoS | 1 | P(F) | "Facebook" OR "Twitter" OR "social network" OR "social media" |
|  | 2 | P(C) | "social media" OR "social networking" |
|  | 3 | F(F) | "Prediction" OR "predicting" OR "detection" OR "detecting" OR "Machine learning" OR "text mining" OR "linguistic style" OR "sentiment analysis" OR "semantic analysis" OR "natural language processing" OR "algorithms" |
|  | 4 | F(C) | "machine learning" OR "natural language processing" OR "sentiment analysis" OR "semantics" OR "algorithms" |
|  | 5 | O(F) | "Depression" OR "Depressive Symptoms" OR "Mood disorder" OR "depressive disorder" |
|  | 6 | O(C) | "depressive disorder" OR "depression" |
|  |  | Filter | English, publication year 2008-2023 |
|  | 7 | All | (#1 OR # 2) AND (#3 OR #4) AND (#5 OR #6) |
|  |  |  | ((ALL=("Facebook" OR "Twitter" OR "social network" OR "social media")) AND ALL=("Prediction" OR "predicting" OR "detection" OR "detecting" OR "Machine learning" OR "text mining" OR "linguistic style" OR "sentiment analysis" OR "semantic analysis" OR "natural language processing" OR "algorithms")) AND ALL=("Depression" OR "Depressive Symptoms" OR "Mood disorder" OR "depressive disorder") and 2023 or 2022 or 2021 or 2020 or 2019 or 2018 or 2017 or 2016 or 2015 or 2008 or 2009 or 2010 or 2011 or 2012 or 2013 or 2014 (Publication Years) and English (Languages) |
|  |  |  |  |
| IEEE explore | 1 | P(F) | - |
|  | 2 | P(C) | "social media" OR "social networking" |
|  | 3 | F(F) | - |
|  | 4 | F(C) | "machine learning" OR "natural language processing" OR "sentiment analysis" OR "semantics" OR "algorithms" |
|  | 5 | O(F) | - |
|  | 6 | O(C) | "depressive disorder" OR "depression" |
|  |  | Filter | Publication year 2008-2023 |
|  | 7 | All | ("All Metadata":"social media" OR "All Metadata":"social networking") AND ("All Metadata":"machine learning" OR "All Metadata":"natural language processing" OR "All Metadata":"algorithms" OR "All Metadata":"sentiment analysis" OR "All Metadata": "semantics ") AND ("All Metadata":"depressive disorder" OR "All Metadata": "depression" |
|  |  |  |  |
| ACM | 1 | P(F) | - |
|  | 2 | P(C) | "social media" OR "social networking" |
|  | 3 | F(F) | - |
|  | 4 | F(C) | "machine learning" OR "natural language processing" OR "sentiment analysis" OR "semantics" OR "algorithms" |
|  | 5 | O(F) | - |
|  | 6 | O(C) | "depressive disorder" OR "depression" |
|  |  | Filter | Publication year 2008-2023 |
|  | 7 | All | [[All: "social media"] OR [All: "social networking"]] AND [[All: "machine learning"] OR [All: "natural language processing"] OR [All: "sentiment analysis"] OR [All: "semantics"] OR [All: "algorithms"]] AND [[All: "depressive disorder"] OR [All: "depression"]] |

P (F) = Population (Free text), P (C) = Population (Control text), F (F) = prediction factors (Free text), F (C) = Prediction factor (Control text), O (F) = Outcome (Free text), O (C) = Outcome (Control text).
