# Supplementary material for: Text-Based Depression Prediction on Social Media Using Machine Learning: Systematic Review and Meta-Analysis
Source: J Med Internet Res. 2025 Apr 11;27:e59002. doi: 10.2196/59002 (PMC12032503; doi:10.2196/59002)
Supplement: Multimedia Appendix 3 [file jmir_v27i1e59002_app3.docx]

Supplementary Table 2. List of Included Studies

| **#** | **Study** |
| --- | --- |
|  |  |
| 1 | Aldarwish, M. M., & Ahmad, H. F. (2017). Predicting Depression Levels Using Social Media Posts. Paper presented at the Proceedings - 2017 IEEE 13th International Symposium on Autonomous Decentralized Systems, ISADS 2017. |
| 2 | Alsagri, H. S., & Ykhlef, M. (2020). Machine learning-based approach for depression  detection in twitter using content and activity features. IEICE transactions on  Information and systems, E103D (8), 1825-1832.  doi:10.1587/transinf.2020EDP7023 |
| 3 | Asad, N. A., Mahmud Pranto, M. A., Afreen, S., & Islam, M. M. (2019). Depression Detection by Analyzing Social Media Posts of User. Paper presented at the 2019 IEEE International Conference on Signal Processing, Information, Communication and Systems, SPICSCON 2019. |
| 4 | Cheng, Q., Li, T. M., Kwok, C.-L., Zhu, T., & Yip, P. S. (2017). Assessing Suicide Risk and Emotional Distress in Chinese Social Media: A Text Mining and Machine Learning Study. Journal of medical internet research, 19(7), 1. doi:https://doi.org/10.2196/jmir.7276 |
| 5 | De Choudhury, M., Gamon, M., Counts, S., & Horvitz, E. (2013). Predicting depression via social media. Paper presented at the Proceedings of the 7th International Conference on Weblogs and Social Media, ICWSM 2013. |
| 6 | Fatima, B., Amina, M., Nachida, R., & Hamza, H. (2020). A Mixed Deep Learning Based Model to Early Detection of Depression. Journal of web engineering, 19(3-4), 429-455. doi:10.13052/jwe1540-9589.19344 |
| 7 | Gupta, B., Pokhriyal, N., & Gola, K. K. (2022). Detecting Depression in Reddit Posts using Hybrid Deep Learning Model LSTM-CNN. Paper presented at the Proceedings of International Conference on Technological Advancements in Computational Sciences, ICTACS 2022. |
| 8 | Jagtap, N., Shukla, H., Shinde, V., Desai, S., & Kulkarni, V. (2021). Use of Ensemble Machine Learning to Detect Depression in Social Media Posts. Paper presented at the Proceedings of the 2nd International Conference on Electronics and Sustainable Communication Systems, ICESC 2021. |
| 9 | Wu, J., Wu, X., Hua, Y., Lin, S., Zheng, Y., & Yang, J. (2023). Exploring Social Media for Early Detection of Depression in COVID-19 Patients. Paper presented at the Proceedings of the ACM Web Conference 2023, Austin, TX, USA. https://doi.org/10.1145/3543507.3583867 |
| 10 | Ríssola, E. A., Bahrainian, S. A., & Crestani, F. (2019, 2019//). Anticipating Depression Based on Online Social Media Behaviour. Paper presented at the Flexible Query Answering Systems, Cham. |
| 11 | Ríssola, E. A., Bahrainian, S. A., & Crestani, F. (2020). A Dataset for Research on Depression in Social Media. Paper presented at the Proceedings of the 28th ACM Conference on User Modeling, Adaptation and Personalization, Genoa, Italy. https://doi.org/10.1145/3340631.3394879 |
| 12 | Yohapriyaa, M., & Uma, M. (2022). Multi-variant Classification of Depression Severity Using Social Media Networks Based on Time Stamp. In Lecture Notes on Data Engineering and Communications Technologies (Vol. 101, pp. 553-564). |
| 13 | Wu, M. Y., Shen, C. Y., Wang, E. T., & Chen, A. L. P. (2020). A deep architecture for depression detection using posting, behavior, and living environment data. Journal of intelligent information systems, 54(2), 225-244. doi:10.1007/s10844-018-0533-4 |
| 14 | Wongkoblap, A., Vadillo, M. A., & Curcin, V. (2018). A multilevel predictive model for detecting social network users with depression. Paper presented at the Proceedings - 2018 IEEE International Conference on Healthcare Informatics, ICHI 2018. |
| 15 | Wang, X., Zhang, C., Ji, Y., Sun, L., Wu, L., & Bao, Z. (2013). A depression detection model based on sentiment analysis in micro-blog social network. Paper presented at the Lecture Notes in Computer Science (including subseries Lecture Notes in Artificial Intelligence and Lecture Notes in Bioinformatics). |
| 16 | Victor, D. B., Kawsher, J., Labib, M. S., & Latif, S. (2020). Machine Learning Techniques for Depression Analysis on Social Media- Case Study on Bengali Community. Paper presented at the Proceedings of the 4th International Conference on Electronics, Communication and Aerospace Technology, ICECA 2020. |
| 17 | Tong, L., Liu, Z., Jiang, Z., Zhou, F., Chen, L., Lyu, J., . . . Zhou, H. (2022). Cost-sensitive Boosting Pruning Trees for depression detection on Twitter. IEEE transactions on affective computing, 1-1. doi:10.1109/TAFFC.2022.3145634 |
| 18 | Tlachac, M. L., & Rundensteiner, E. (2020). Screening for Depression with Retrospectively Harvested Private Versus Public Text. Ieee journal of biomedical and health informatics, 24(11), 3326-3332. doi:10.1109/JBHI.2020.2983035 |
| 19 | Tejaswini, V., Babu, K. S., & Sahoo, B. (2022). Depression Detection from Social Media Text Analysis using Natural Language Processing Techniques and Hybrid Deep Learning Model. ACM Trans. Asian Low-Resour. Lang. Inf. Process. doi:10.1145/3569580 |
| 20 | Sudhishna, K. S., Kumar, C. S., Kishore, B. S., & Arun, A. (2023, 14-16 June 2023). Comparative Analysis of Different Machine Learning Algorithms to Predict Depression. Paper presented at the 2023 International Conference on Sustainable Computing and Smart Systems (ICSCSS). |
| 21 | Stankevich, M., Latyshev, A., Kuminskaya, E., Smirnov, I., & Grigoriev, O. (2019). Depression detection from social media texts. Paper presented at the CEUR Workshop Proceedings. |
| 22 | Shekerbekova, S., Yerekesheva, M., Tukenova, L., Turganbay, K., Kozhamkulova, Z., & Omarov, B. (2021). Applying Machine Learning to Detect Depression-Related Texts on Social Networks. Paper presented at the Communications in Computer and Information Science. |
| 23 | Saini, G., Yadav, N., & Kamath S, S. (2022). Ensemble Neural Models for Depressive Tendency Prediction Based on Social Media Activity of Twitter Users. Paper presented at the Lecture Notes in Electrical Engineering. |
| 24 | Ricard, B. J., Marsch, L. A., Crosier, B., & Hassanpour, S. (2018). Exploring the Utility of Community-Generated Social Media Content for Detecting Depression: An Analytical Study on Instagram. Journal of medical internet research, 20(12). doi:10.2196/11817 |
| 25 | Narynov, S., Mukhtarkhanuly, D., Omarov, B., Kozhakhmet, K., & Omarov, B. (2020). Machine learning approach to identifying depression related posts on social media. Paper presented at the International Conference on Control, Automation and Systems. |
| 26 | Mann, P., Paes, A., & Matsushima, E. H. (2020). See and read: Detecting depression symptoms in higher education students using multimodal social media data. Paper presented at the Proceedings of the 14th International AAAI Conference on Web and Social Media, ICWSM 2020. |
| 27 | Kumar, S. K., Dinesh, N., & Nitha, L. (2022). Depression Detection in Twitter Tweets Using Machine Learning Classifiers. Paper presented at the Proceedings - 2022 2nd International Conference on Interdisciplinary Cyber Physical Systems, ICPS 2022. |
| 28 | Kumar, A., Pratihar, V., Kumar, S., & Abhishek, K. (2021). Predicting Depression by Analysing User Tweets. Paper presented at the Lecture Notes in Electrical Engineering. |
| 29 | Kour, H., & Gupta, M. K. (2022). Hybrid LSTM-TCN Model for Predicting Depression using Twitter Data. Paper presented at the 2022 17th International Conference on Control, Automation, Robotics and Vision, ICARCV 2022. |
| 30 | Kour, H., & Gupta, M. K. (2022). Predicting the language of depression from multivariate twitter data using a feature-rich hybrid deep learning model. Concurrency and computation-practice & experience, 34(24). doi:10.1002/cpe.7224 |
| 31 | Kabir, M., Ahmed, T., Hasan, M. B., Laskar, M. T. R., Joarder, T. K., Mahmud, H., & Hasan, K. (2023). Deptweet: A typology for social media texts to detect depression severities. Computers in human behavior, 139. doi:10.1016/j.chb.2022.107503 |
| 32 | Islam, M. R., Kamal, A. R. M., Sultana, N., Islam, R., Moni, M. A., & ulhaq, A. (2018, 8-9 Feb. 2018). Detecting Depression Using K-Nearest Neighbors (KNN) Classification Technique. Paper presented at the 2018 International Conference on Computer, Communication, Chemical, Material and Electronic Engineering (IC4ME2). |
| 33 | Dey, S., Nawshin, A. T., Hossain, A., & Rahman, R. M. (2022, 12-14 Oct. 2022). Machine Learning Combined with PHQ-9 for Analyzing Depression in Bangladeshi Metropolitan Areas. Paper presented at the 2022 IEEE 11th International Conference on Intelligent Systems (IS). |
| 34 | Reece, A. G., Reagan, A. J., Lix, K. L. M., Dodds, P. S., Danforth, C. M., & Langer, E. J. (2017). Forecasting the onset and course of mental illness with Twitter data. Scientific Reports, 7(1), 13006. doi:10.1038/s41598-017-12961-9 |
| 35 | Mariñelarena-Dondena, Luciana; Ferretti, Edgardo; Maragoudakis, Manolis; Sapino, Maximiliano Emanuel; Errecalde, Marcelo Luis; Predicting Depression: a comparative study of machine learning approaches based on language usage; Center for Academic Studies in Neuropsychology; Neuropsychology Notebooks; eleven; 3; 12-2017; 42-54 |
| 36 | Schwartz, H. A., Eichstaedt, J., Kern, M., Park, G., Sap, M., Stillwell, D., . . . Ungar, L. (2014). Towards assessing changes in degree of depression through facebook. Paper presented at the Proceedings of the workshop on computational linguistics and clinical psychology: from linguistic signal to clinical reality. |
