# Supplementary material for: Text-Based Depression Prediction on Social Media Using Machine Learning: Systematic Review and Meta-Analysis
Source: J Med Internet Res. 2025 Apr 11;27:e59002. doi: 10.2196/59002 (PMC12032503; doi:10.2196/59002)
Supplement: Multimedia Appendix 5 [file jmir_v27i1e59002_app5.docx]

Supplementary Table 3. Summary of the Characteristics of the Included Studies

| **Author, Year** | **Publication** | **Age/mean & SD/ median** | **Female (%)** | **Sample size** | **Data points** | **Social media** | **Type of social media** | **Features** | **Type of features** | **Measurement** | **ML Algorithm** | **Machine learning approach** | **Model validation** |
| --- | --- | --- | --- | --- | --- | --- | --- | --- | --- | --- | --- | --- | --- |
|  |  |  |  |  |  |  |  |  |  |  |  |  |  |
| Cheng,2017 | Journal | 18 and above | NA | 974 | 349374 | Sina Weibo | Public | Second person plural, work-related, Achieve-related | Linguistic | DASS | SVM | Shallow | Leave-one-out cross-validation |
| De Choudhury, 2013 | Proceedings | NA | 49 | 476 | NA | Twitter | Public | Engagement, ego-network, emotion, linguistic style, dep. Language, demographics | Linguistic, activity, demographics | CES-D, BDI | SVM (RBF kernel) | Shallow | 10-fold cross-validation |
| Kabir, 2022 | Journal | NA | NA | 344657 | 45000 | Twitter | Public | NA | NA | PHQ-9 | DistilBERT | Deep | Hold out validation |
| Mann, 2020 | Proceedings | 18 and above | 62 | 221 | NA | IG | Public | Pronouns, social words, biological process | Linguistic | BDI | Elmo | Deep | 10-fold cross-validation |
| Ricard, 2018 | Journal | mean 26.7, SD 7.29 | 69 | 749 | NA | IG | Public | Number of posts, regularization | Activity | PHQ-8 | LR | Shallow | 20-fold cross-validation |
| Tlachac, 2022 | Journal | NA | NA | 105 | 45908 | Twitter | Public | Word category frequency, part of speech tags, sentiment, volume | Linguistic | PHQ-9 | LR | Shallow | 5-fold cross-validation |
| Wongkoblap, 2018 | Proceedings | median 22 | 59 | 4235 | 147470 | FB | Private | Demographics, activities, posting time, text meaning | Demographics, activity | CES-D | SVM (RBF kernel) | Shallow | 10-fold cross-validation |
| Wu, 2018 | Journal | median 21 | 57 | 1294 | 873524 | FB | Private | Content-based features, behavior features | Linguistic, activity | CES-D | DNN | Deep | 10-fold cross-validation |
| Islam, 2018 | Proceedings | NA | NA | NA | 7145 | FB | Public | Emotional, temporal, language | Linguistic, temporal | NA | Course KNN | Shallow | 10-fold cross-validation |
| Rissola, 2020 | Proceedings | NA | NA | NA | 7000 | Reddit | Public | Contextual language | Linguistic | DSM criteria | LR | Shallow | Binary classification validation |
| Shekerbokova, 2021 | Proceedings | NA | NA | NA | 64000 | NA | NA | NA | NA | NA | SVM | Shallow | NA |
| Wang, 2013 | Proceedings | NA | NA | 180 | 6014 | Sina Micro-blog | Public | Sentence polarity, quantity of first person pronouns, ratio of the first person to prural pronouns, quantity of emotions, ratio of positive emotions to negative emotions, times of mentioning others, times of being forwarded, times of being commented, percentage of original micro-blogs, active period | Temporal, activity, linguistic | Psychologist diagnosis | BayesNet | Shallow | 10-fold cross-validation |
| Wu, 2023 | Proceedings | NA | NA | 10656 | 10488061 | Twitter | Public | COVID Infection time, depression time, daily tweets aggregation | Temporal | NA | DNN | Deep | NA |
| Dey, 2022 | Proceedings | NA | 25 | NA | 1778 | FB | Private | NA | NA | PHQ-9 | RF | Shallow | 5-fold cross-validation |
| Kour, 2022 | Proceedings | NA | NA | NA | 11879 | Twitter | Public | NA | NA | NA | LSTM-TCN | Deep | NA |
| Jagtap, 2021 | Proceedings | NA | NA | NA | 20000 | NA | NA | NA | NA | NA | KNN (blending) | Shallow | NA |
| Narynov, 2020 | Proceedings | NA | NA | NA | 85800 | Vkontakte | Public | NA | NA | NA | RF | Shallow | 5-fold cross-validation |
| Alsagri, 2020 | Journal | NA | NA | 500 | 1000000 | Twitter | Public | User activity, sentiments, synonyms, use of words, self-center | Linguistic, activity | NA | SVM-Linear | Shallow | 10-fold cross-validation |
| Kour, 2022 | Journal | NA | NA | NA | 11879 | Twitter | Public | NA | NA | NA | CNN-LSTM | Deep | 10-fold cross-validation |
| Kumar, 2022 | Proceedings | NA | NA | NA | 5000 | Twitter | Public | NA | NA | NA | DT | Shallow | NA |
| Sudhishna, 2023 | Proceedings | NA | NA | NA | NA | Twitter | Public | NA | NA | NA | SVM | Shallow | NA |
| Ardawish, 2017 | Proceedings | NA | NA | NA | 6773 | Twitter, FB, LiveJournal | Both | NA | NA | DSM-IV, BDI | NB | Shallow | NA |
| Gupta, 2022 | Proceedings | NA | NA | NA | 231943 | Reddit | Public | NA | NA | NA | LSTM-CNN | Deep | 5-fold cross-validation |
| Victor, 2020 | Proceedings | NA | NA | NA | 35000 | Twitter, FB | Both | NA | NA | NA | LR | Shallow | NA |
| Kumar, 2021 | Proceedings | NA | NA | NA | 3223 | Twitter | Public | Depression language, word count, lexicon score | Linguistic | NA | SVM | Shallow | NA |
| Asad, 2019 | Proceedings | NA | NA | 150 | NA | Twitter | Public | NA | NA | BDI-II | SVM+NB | Shallow | NA |
| Stankevich, 2019 | Proceedings | 24.88±6.47 | 68.53 | 1020 | 15238 | Vkontakte | Public | Psycholinguistic markers, bigrams, unigrams, dictionaries | Linguistic | BDI-II | SVM+PM-r | Shallow | 4-fold cross-validation |
| Tong 2022 | Journal | NA | NA | NA | NA | Twitter | Public | User profile, social interaction, linguistic features | Linguistic, activity, demographic | NA | CBPT | Shallow | NA |
| Vankayala, 2022 | Journal | NA | NA | NA | 6164 | Twitter | Public | NA | NA | NA | FCL | Deep | NA |
| Boumahdi, 2020 | Journal | NA | NA | 887 | 500000 | Reddit | Public | NA | NA | NA | CNN-BiLSTM | Deep | NA |
| Yohapriyaa 2022 | Proceedings | NA | NA | 50 | NA | Twitter | Public | NA | NA | NA | CNN-LSTM | Deep | NA |
| Saini 2022 | Proceedings | NA | NA | NA | 1600000 | Twitter | Public | Depression-indicative words, day and night window | Linguistic, temporal | NA | SVM | Shallow | NA |
| Reece, 2017 | Journal | NA | NA | 204 | 279951 | Twitter | Public | Total tweets/user/day, happiness of tweet language, parts of speech, semantic category | Activity, linguistic | CES-D | RF | Shallow | 5-fold cross-validation |
| Rissola 2019 | Proceedings | NA | NA | 1707 | 1076582 | Reddit | Public | Function words, Word counts | Linguistic | NA | LR | Shallow | NA |
| Mariñelarena-Dondena 2017 | Journal | NA | NA | NA | 486 | Reddit | Public | Nouns, pronouns, verbs | Linguistic | NA | Deep learning +SMOTE | Deep | NA |
| Schwartz 2014 | Proceedings | 24.8 | 57 | 28,749 | 69,917,624 | FB | Private | Topics, sentiments, n grams | Linguistic | IPIP | Regression | Shallow | NA |

FB = Facebook, DASS = depression, anxiety, stress scale, CES-D = center for the epidemiologic studies depression scale, BDI = beck depression inventory, PHQ = patient health questionnaire, SVM = support vector machine, RBF = radial basis function, BERT = bidirectional encoder representations from transformers, Elmo = Embedding from Language Model, LR = linear regression, DNN = deep neural network, SMOTE = Synthetic minority oversampling technique, IPIP = international personality item pool
