# Supplementary material for: Text-Based Depression Prediction on Social Media Using Machine Learning: Systematic Review and Meta-Analysis
Source: J Med Internet Res. 2025 Apr 11;27:e59002. doi: 10.2196/59002 (PMC12032503; doi:10.2196/59002)

**Figure S1.** A forest plot of the influence of demographic features in predicting depression.


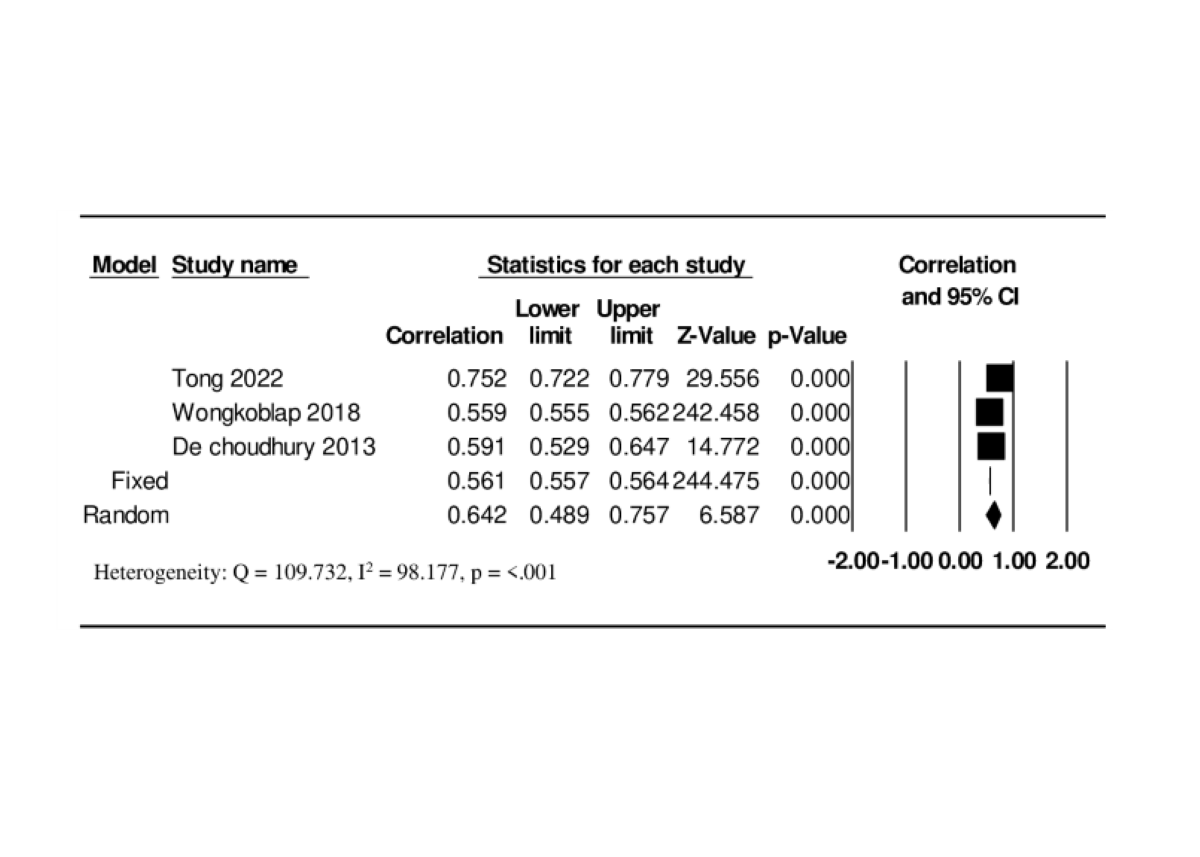


**Figure S2.** A forest plot of the influence of social media activity features in predicting depression.


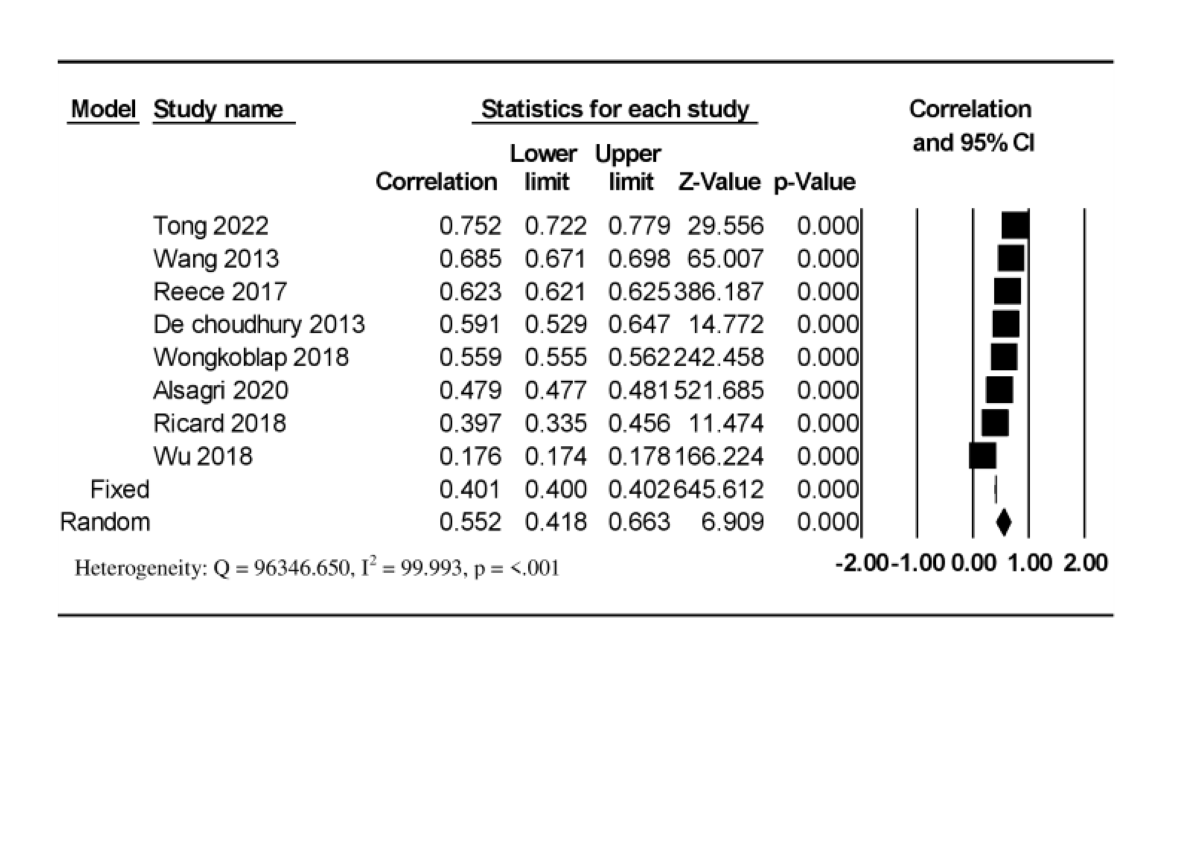


**Figure S3.** A forest plot of the influence of language features in predicting depression.


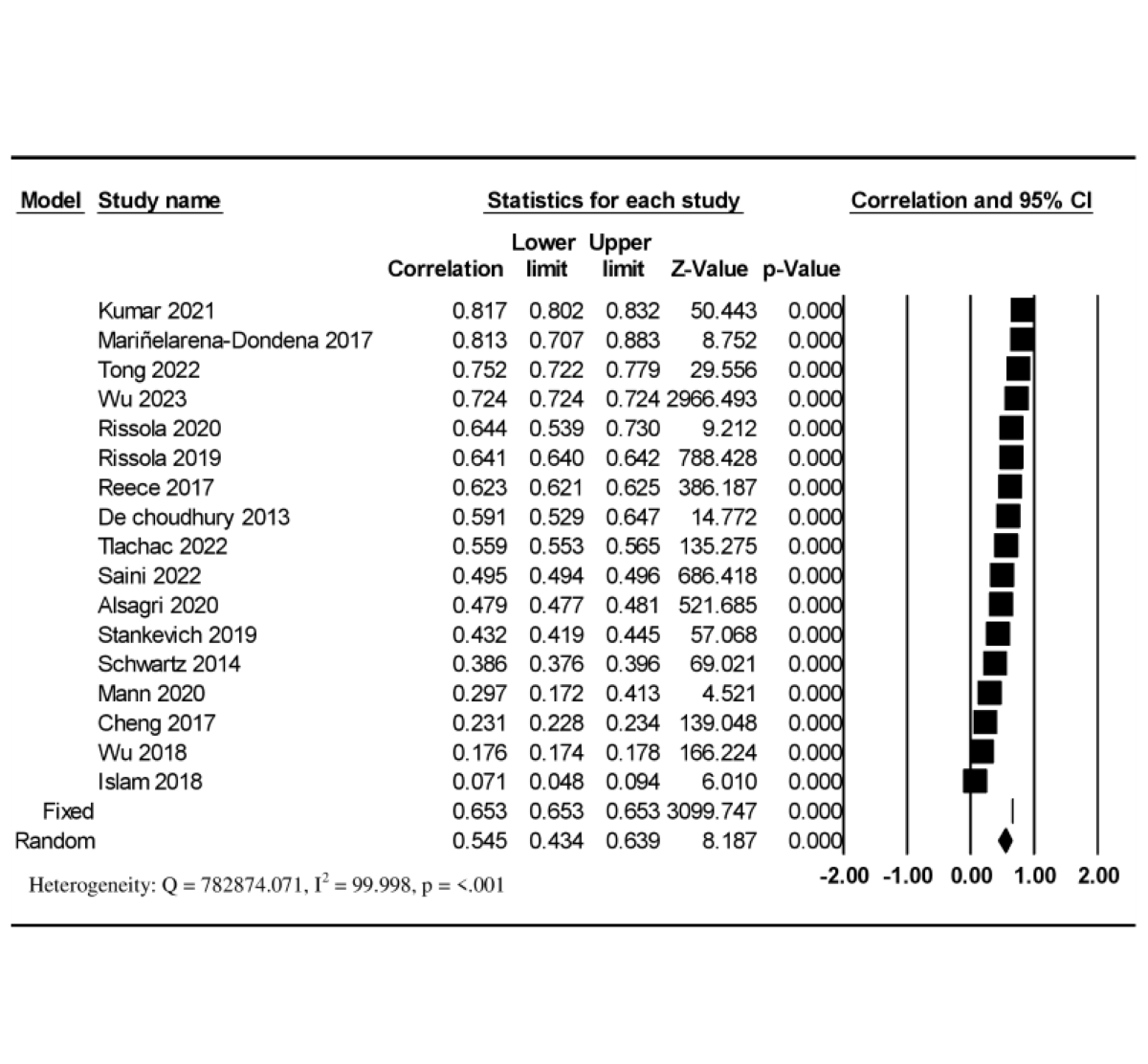


**Figure S4.** A forest plot of the influence of temporal features in predicting depression.


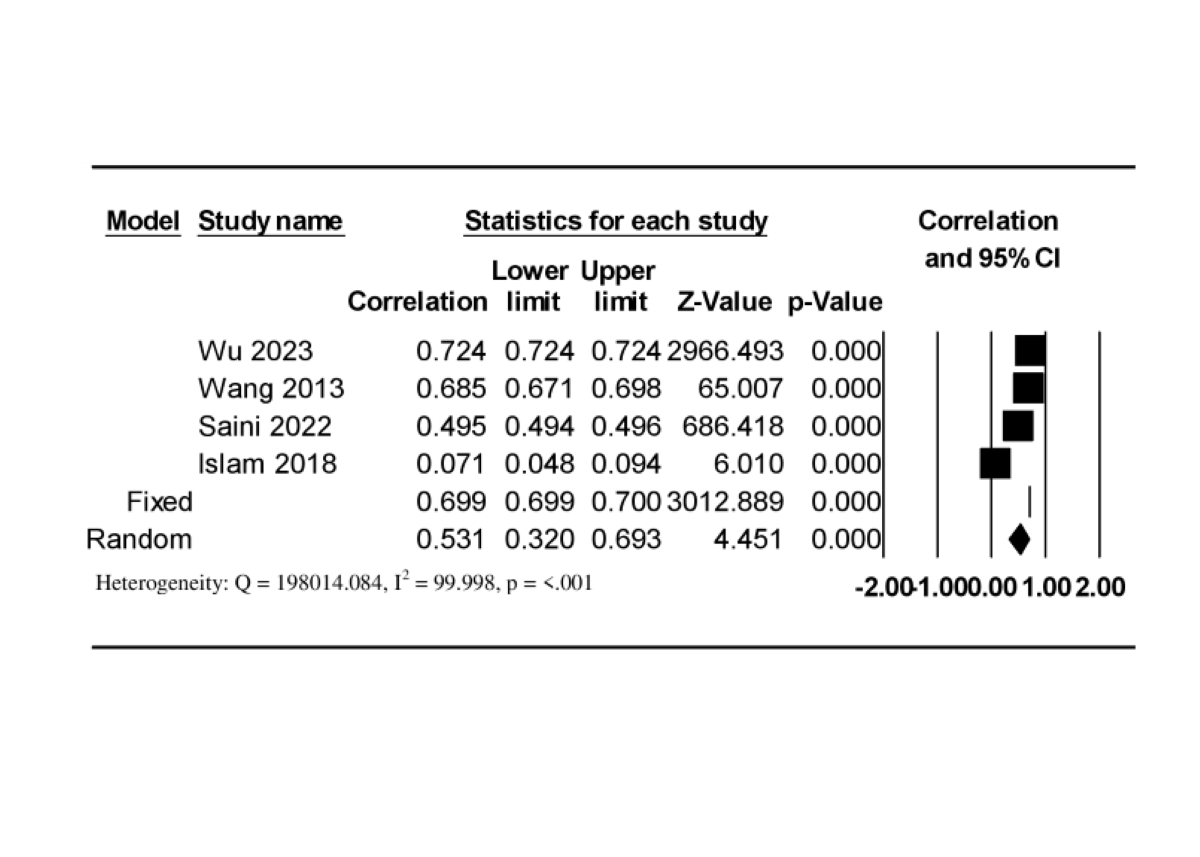

Supplement: Multimedia Appendix 6 [file jmir_v27i1e59002_app6.docx]
